# STING agonist diABZI confers protection against swine acute diarrhea syndrome coronavirus in neonatal mice by activating antiviral immunity

**DOI:** 10.1128/jvi.01703-25

**Published:** 2025-12-29

**Authors:** Yuying Li, Wei Chen, Xinyu Zhang, Jiyong Zhou, Yanqing Hu, Yimin Zhou, Tian Lan, Haixin Huang, Lulu Xie, Yan Qin, Lin Zhou, Wenchao Sun, HuiJun Lu

**Affiliations:** 1 MOA Key Laboratory of Animal Virology, Zhejiang University Center for Veterinary Sciences 12377, Hangzhou, China; 2 Changchun Veterinary Research Institute, Chinese Academy of Agricultural Sciences 595703 , Changchun, China; 3 Wenzhou Key Laboratory for Virology and Immunology, Institute of Virology, Wenzhou University 26495, Wenzhou, China; 4 Agricultural College, Yanbian University 118411, Yanji, China; University of North Carolina at Chapel Hill, Chapel Hill, North Carolina, USA

**Keywords:** swine acute diarrhea syndrome coronavirus (SADS-CoV), STING pathway, interferon, innate immune response

## Abstract

The recently identified alphacoronavirus swine acute diarrhea syndrome coronavirus (SADS-CoV) has a high fatality rate in neonatal piglets. Currently, no vaccines or treatment strategies for SADS-CoV infection are available. The stimulator of interferon genes (STING) pathway plays a critical role in initiating innate immune responses against RNA viral infections; however, its role in host defense against SADS-CoV infection remains unexplored. We assessed the pathogenicity of SADS-CoV in 3-day-old, 7-day-old, and 3-week-old mice, revealing striking age-dependent susceptibility—a pattern mirroring clinical observations in piglets. Additionally, SADS-CoV infection activated the STING-dependent pathway, which resulted in significant interferon responses in infected mice. *In vitro* experimental findings confirmed that STING pathway activation inhibited SADS-CoV replication by modulating the NF-κB and IRF3 signaling pathways and mediating the production of inflammatory cytokines, which underscores the importance of the STING pathway in antiviral defense mechanisms. *In vivo* studies revealed that the STING inhibitor C176 significantly promoted viral replication, whereas activation of the STING pathway using the STING agonist diABZI increased antiviral immune responses and reduced viral replication. Notably, diABZI protected mice from SADS-CoV infection by reducing viral replication through mechanisms involving both type I interferon-dependent and -independent pathways. These results represent the first demonstration of the *in vivo* therapeutic efficacy of pharmacological STING activation against SADS-CoV. These findings demonstrate that the STING pathway serves as a critical regulator of host defense against SADS-CoV and suggest that STING-targeted intervention has therapeutic potential.

**IMPORTANCE:**

Swine acute diarrhea syndrome coronavirus (SADS-CoV) is an emerging zoonotic pathogen with significant implications for veterinary and public health; it has a high mortality rate in piglets and the potential for cross-species transmission. Currently, there are no approved vaccines or specific antiviral agents available for this pathogen. In this study, we demonstrated that the stimulator of interferon genes (STING) pathway serves as a critical mediator of host defense against SADS-CoV infection. STING activation inhibits viral replication by coordinating interferon responses and modulating NF-κB/IRF3 signaling, and its inhibition exacerbates infection. Importantly, pharmacological activation of the STING pathway using the agonist diABZI significantly inhibited viral replication *in vivo* in a STING-dependent manner, with contributions from both type I interferon-dependent and -independent antiviral mechanisms, highlighting its therapeutic potential. These results advance our understanding of antiviral defense strategies against SADS-CoV and identify STING pathway regulation as a viable therapeutic approach for this emerging pathogen.

## INTRODUCTION

Swine acute diarrhea syndrome coronavirus (SADS-CoV) is a recently identified alphacoronavirus that was discovered in Guangdong Province, China (1). The symptoms of SADS-CoV infection, such as severe diarrhea, vomiting, and dehydration, are similar to those of other coronavirus infections in pigs, and the mortality rate of SADS-CoV is 35% in piglets under 10 days of age (2, 3). SADS-CoV infection is associated with a mortality rate of up to 90% in piglets under 5 days of age. (4, 5). Unlike other coronaviruses, SADS-CoV can infect a variety of cell lines *in vitro*, including those derived from bats, chickens, humans, hamsters, gerbils, mice, nonhuman primates, pigs, and rats (6). These results indicate that its infectivity is not confined to swine, which highlights the potential risk of cross-species transmission to humans (7). Given its broad species tropism and high lethality in neonates, there is an urgent need for effective therapeutic strategies and accessible animal models to dissect host-pathogen interactions. Current *in vivo* studies of SADS-CoV pathogenesis rely primarily on pig models; however, their high cost and stringent biosafety requirements limit their broad application. Although mouse models offer a more accessible and cost-effective alternative, their use in SADS-CoV research remains underexploited, hindering mechanistic insights into viral pathogenesis (8, 9). In this study, we established a robust neonatal mouse model that recapitulates key clinical features of SADS-CoV infection—including watery diarrhea, weight loss, and neuroinflammation—and enables the detailed investigation of immune responses.

STING is a crucial innate immune signaling molecule that was initially identified as a sensor for DNA viruses and cytosolic DNA (10, 11), and type I interferon responses are activated through the cGAS-STING pathway (12). However, accumulating evidence indicates that the STING pathway also contributes to host defense against certain RNA viruses. For example, during influenza A virus or dengue virus infection, mitochondrial damage leads to the leakage of mitochondrial DNA (mtDNA) into the cytosol, which activates cGAS-STING and triggers interferon responses (13, 14). Similarly, impaired STING signaling increases susceptibility to vesicular stomatitis virus (15). Despite these advances, the role of STING in coronavirus infections is largely unexplored. Moreover, whether pharmacological modulation of STING can confer protection against highly pathogenic coronaviruses such as SADS-CoV *in vivo* remains unknown.

Here, we characterize the age-dependent pathogenicity of SADS-CoV in a neonatal mouse model and define the critical role of the STING pathway in antiviral defense. We demonstrate that STING activation restricts SADS-CoV replication via interferon-dependent mechanisms and that pharmacological targeting of STING with diABZI confers protection *in vivo*, providing proof-of-concept for host-directed therapeutic strategies against this emerging pathogen.

## RESULTS

### Neonatal mice are susceptible to SADS-CoV infection

**Fig 1:**
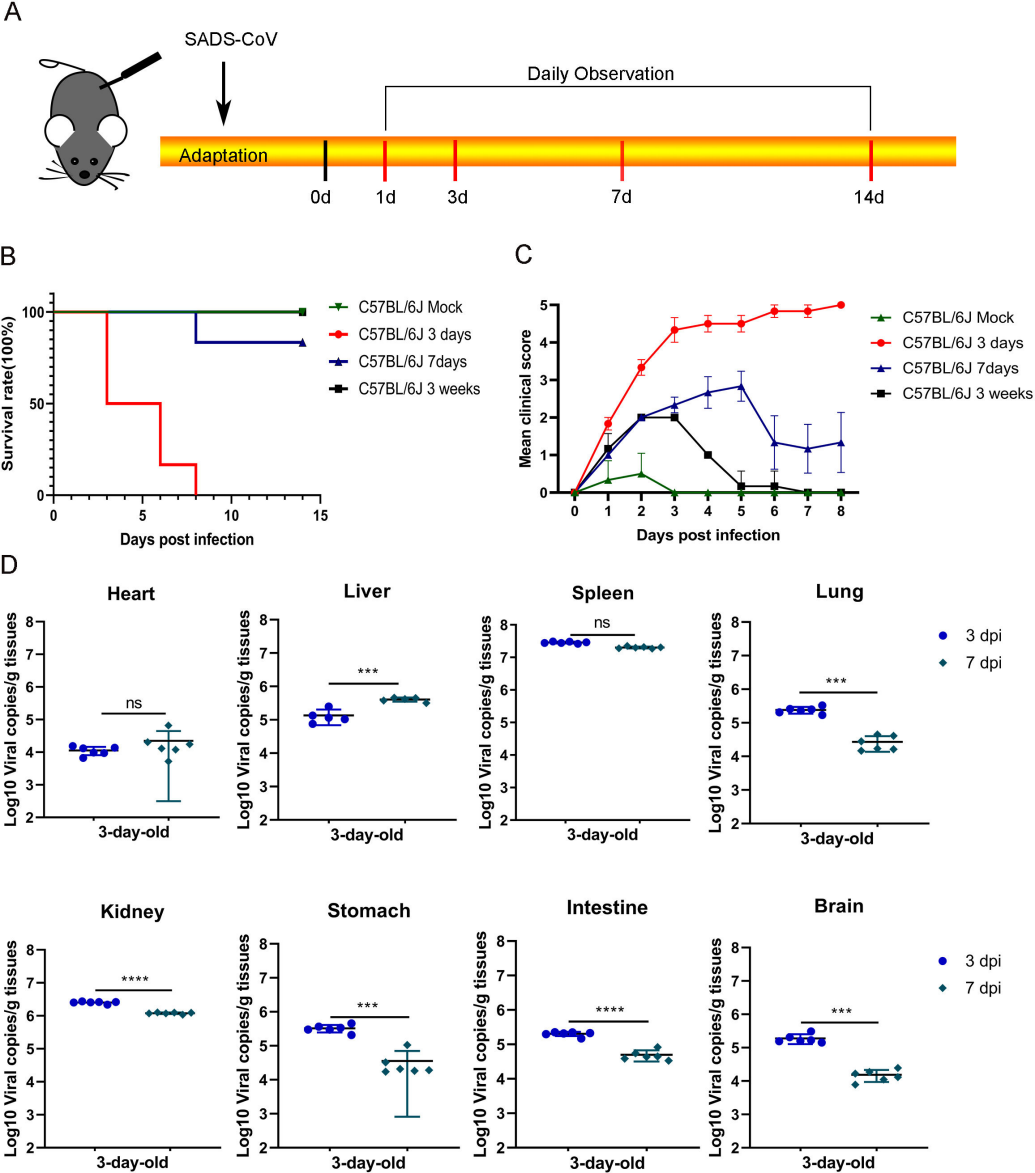
SADS-CoV infection has high lethality in C57BL/6J mice. Three-day-old, 7-day-old, and 3-week-old mice were infected with SADS-CoV, and 3-day-old infected mice were used as a control group (*n* = 6). (**A**) Newborn C57BL/6J mice were inoculated with SADS-CoV via intraperitoneal injection. (**B**) Daily monitoring of survival rate. (**C**) Clinical scores were monitored daily, with clinical scoring based on Table S1. (**D**) Viral RNA in tissues collected from infected mice at 3 and 7 dpi was quantified using RT-qPCR. Data (mean ± SD) are from three independent triplicate experiments. ns, not significant; *P < 0.05; **P < 0.01; ***P < 0.001; ****P < 0.0001.

The pathogenicity of SADS-CoV in wild-type (WT) C57BL/6J mice was investigated by infecting 3-day-old, 7-day-old, and 3-week-old mice. Each group consisted of six mice of each age, which were intraperitoneally injected with the virus at a dose of 10^6^ 50% tissue culture infectious dose (TCID_50_) or with DMEM as a control. Clinical symptoms were monitored daily for 14 days (Fig. 1A). Furthermore, C57BL/6J mice aged 3 days, 7 days, and 3 weeks were injected with the same dose of SADS-CoV, and their clinical symptoms were monitored daily. Symptoms were classified into five levels on the basis of their clinical manifestation (Table S1). All 3-day-old C57BL/6J mice (*n* = 6) died at 8 days post-infection (dpi), whereas one-sixth of the 7-day-old mice died at the same time point (Fig. 1B). Three-day-old and 7-day-old mice presented severe symptoms post infection, including weight loss, spasms, and death, whereas the 3-week-old mice remained asymptomatic until the end of the experiment (Fig. 1C). To investigate viral replication, viral RNA levels were quantified in tissues from 3-day-old mice by RT-qPCR following euthanasia at different time points (Fig. 1D). The results confirmed the presence of SADS-CoV infection in multiple tissues.

**Fig 2:**
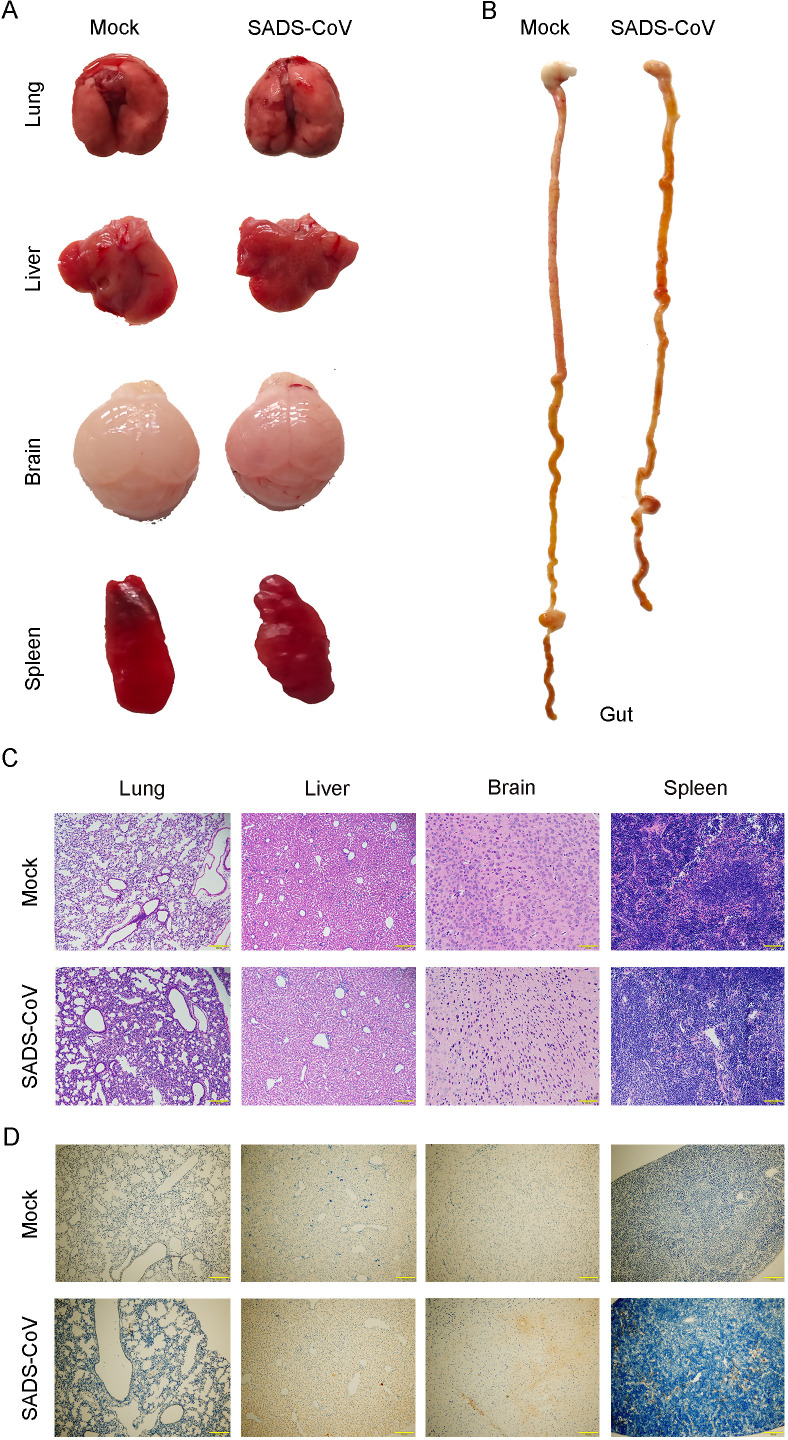
Pathological changes in mice infected with SADS-CoV. At 3 days post-infection, 3-day-old mice (*n* = 6) infected with SADS-CoV and mock-infected controls were euthanized tissue analysis. (**A, B**) Representative gross images of tissues from infected and mock-infected mice: (**A**) lung, liver, brain, and spleen; (**B**) gastrointestinal tract. (**C**) Hematoxylin and eosin (H&E) staining of lung, liver, brain, and spleen tissues to visualize histopathological changes. Scale bar: 100 μm. (**D**) Immunohistochemical analysis of SADS-CoV nucleocapsid (N) protein expression in lung, liver, brain, and spleen tissues.

Next, tissue pathology in SADS-CoV-infected neonatal mice was evaluated. In 3-day-old infected pups, gross inspection revealed pulmonary hemorrhage, multifocal white necrotic foci in the liver, cerebral petechiae, and splenomegaly (Fig. 2A). The stomach and intestines appeared atrophic and were filled with yellowish contents (Fig. 2B). To characterize the histopathological changes, lung, liver, brain, and spleen tissues were subjected to hematoxylin and eosin (H&E) staining. Histological analysis revealed multifocal hepatocellular necrosis with inflammatory cell infiltration in the liver, alveolar wall disruption and interstitial inflammation in the lungs, increased inflammatory cells in the splenic red pulp, and neuronal necrosis in the brain (Fig. 2C). Immunohistochemical detection of the SADS-CoV nucleocapsid (N) protein revealed positive signals in the lung, liver, brain, and spleen at 3 dpi (Fig. 2D), indicating that viral antigen was distributed across multiple tissues. These findings demonstrated that SADS-CoV replicated in various organs and caused significant histopathological damage in neonatal mice.

### SADS-CoV infection in neonatal mice activates the STING pathway

**Fig 3:**
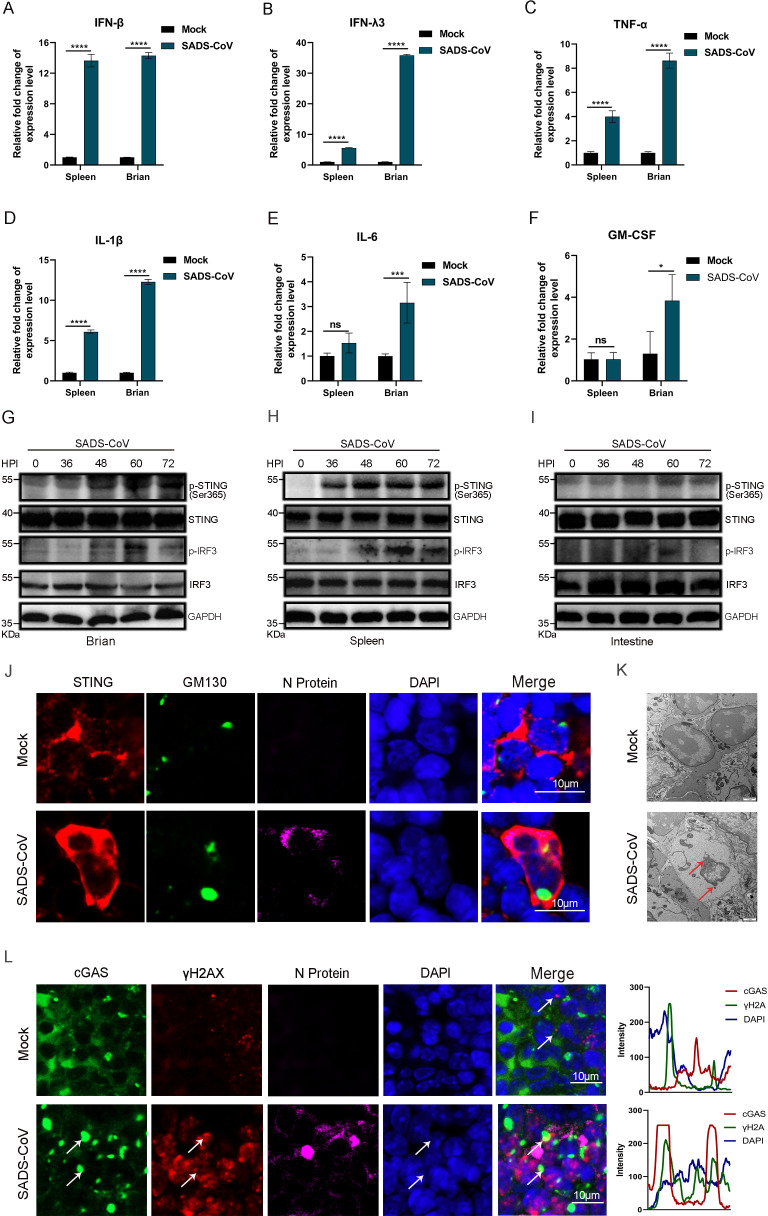
SADS-CoV infection in neonatal mice activates the STING pathway. Three-day-old neonatal mice (*n* = 6) were infected with SADS-CoV or subjected to mock infection with DMEM, followed by euthanasia at different time points. (**A**) Tissue samples from the brain and spleen were collected and analyzed using RT-qPCR. The expression levels of IFN-β (**A**), IFN-λ3 (**B**), TNF-α (**C**), IL-1β (**D**), IL-6 (**E**), and GM-CSF (**F**) were measured. Data (mean ± SD) are from three independent triplicate experiments. ns, not significant; *P < 0.05; **P < 0.01; ***P < 0.001; ****P < 0.0001. (**G–I**) The phosphorylation levels of STING and IRF3 in the brain (**G**), spleen (**H**), and intestine (**I**) were assessed via western blotting. (**J**) Immunofluorescence was used to detect the expression of STING (red), GM130 (green), and SADS-CoV N protein (purple) in the spleen. (**K**) TEM analysis showing the nuclear ultrastructure in the splenic tissues of mice with or without viral infection. Red arrows indicate sites of nuclear leakage. (**L**) Immunofluorescence was used to detect the expression of cGAS (green), γH2AX (red), and SADS-CoV N protein (purple) in the spleen. Scale bar, 10 μm. The fluorescence intensity along the indicated line was analyzed using ImageJ. White arrows indicate quantification regions for fluorescence intensity.

Owing to the high susceptibility of suckling mice to SADS-CoV infection, this model was employed to investigate viral pathogenesis and host immune responses. To elucidate the immune response dynamics following SADS-CoV infection, 3-day-old neonatal mice (*n* = 6) were inoculated intraperitoneally with 10^6^ TCID_50_ virus. Control animals received an equal volume of RPMI 1640 medium. At 48 h post-infection, the mice were euthanized, and brain and spleen tissues were collected for RT-qPCR analysis of the expression of inflammatory cytokines and interferon (Fig. 3A through F). IFN-β was significantly upregulated in both the brain and the spleen, indicating a dominant type I interferon response during SADS-CoV infection (Fig. 3A). The expression of IFN-λ3, a type III interferon, was also markedly elevated in both tissues (Fig. 3B). Tissue-specific analysis revealed increased expression of TNF-α, IL-1β, IL-6, and GM-CSF in the brain (Fig. 3C through F), whereas TNF-α and IL-1β expression was most strongly induced in the spleen (Fig. 3C and D). These results demonstrated that SADS-CoV infection triggered a robust innate immune response in neonatal mice.

To investigate the signaling pathways involved in interferon induction, we examined the cGAS-STING pathway, a key cytosolic DNA sensing mechanism (16). Although SADS-CoV is an RNA virus, its ability to activate this pathway remains unclear. We hypothesized that SADS-CoV might activate interferon production through noncanonical mechanisms, particularly via the cGAS-STING axis. To test this hypothesis, we analyzed the phosphorylation of STING at Ser365 (a key activation marker in mice), a critical marker of STING activation (17), at different time points post-infection. Clear phosphorylation of STING was detected in the brain, spleen, and intestinal tissues (Fig. 3G through I). STING activation requires its translocation from the endoplasmic reticulum to the Golgi apparatus. Immunofluorescence analysis revealed aggregation and distinct subcellular redistribution of STING in infected tissues at 48 h post-infection (Fig. 3J), supporting the activation of the cGAS-STING pathway. To explore the mechanism underlying STING activation, we performed an ultrastructural analysis of splenocytes from infected mice using transmission electron microscopy. Some cells exhibited discontinuities in the nuclear envelope and leakage of nucleoplasmic content, while mitochondrial morphology remained largely intact (Fig. 3K). Although typical viral particles were not observed, possibly because of the timing of sample collection or limited sampling, we further assessed cytosolic DNA accumulation and cGAS activation by immunofluorescence colocalization. Punctate signals of γH2AX, a marker of DNA double-strand breaks, colocalized with cGAS in the cytoplasm, with occasional signals in nuclear regions (Fig. 3L). These findings suggested that SADS-CoV infection might cause genomic instability or DNA leakage, leading to activation of the cGAS-STING signaling axis.

Collectively, these findings provided multilevel evidence that SADS-CoV infection activated the cGAS-STING pathway in neonatal mice and induced a strong interferon-mediated innate immune response.

### STING pathway activation suppresses SADS-CoV replication and modulates inflammatory responses

**Fig 4:**
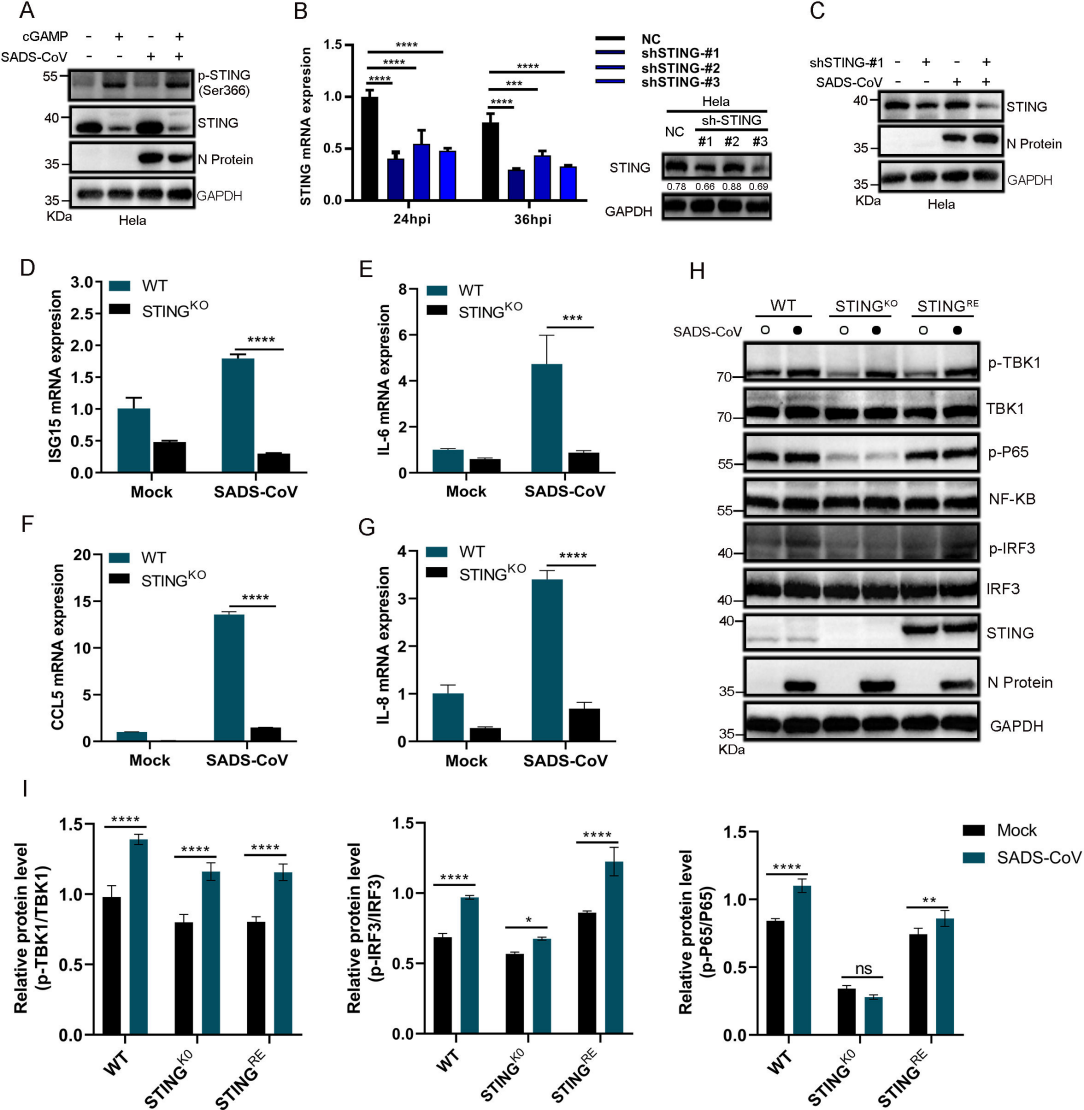
The STING pathway suppresses SADS-CoV replication and modulates inflammatory responses. (**A**) HeLa cells were infected with SADS-CoV (MOI = 1), followed by treatment with either DMSO (control) or 1 μg/μL 2′3′-cGAMP for 8 h. The levels of STING and N were assessed via western blotting. (**B**) HeLa cells expressing shRNAs targeting STING (sh-STING-#1, sh-STING-#2, and sh-STING-#3) or control shRNAs were mock-infected or infected with SADS-CoV (MOI = 1) for 24 or 36 h. The expression levels of STING mRNA and protein were analyzed using qPCR and western blotting, respectively. The STING/GAPDH ratios were quantified using ImageJ. Data (mean ± SD) are from three independent triplicate experiments. ns, not significant; *P < 0.05; **P < 0.01; ***P < 0.001; ****P < 0.0001. (**C**) HeLa and HeLa-sh-STING-#1 cells were mock-infected or infected with SADS-CoV. The levels of STING and N were assessed using western blotting. (**D–G**) Cells or STING-knockout (STING^KO^) cells were mock-infected or infected with SADS-CoV. RNA was extracted and quantified by qPCR to assess the expression of ISG15, IL-6, CCL5, and IL-8 in WT and STING^KO^ cells. Data (mean ± SD) are from three independent triplicate experiments. ns, not significant; *P < 0.05; **P < 0.01; ***P < 0.001; ****P < 0.0001. (**H**) STING-knockout (STING^KO^) cells, cells expressing STING (STING^RE^), and empty vector control (EV Ctrl) cells were infected with SADS-CoV. The phosphorylation levels of P65, IRF3, and TBK1 were analyzed by western blotting. (**I**) The relative levels of phosphorylated and total proteins for TBK1, IRF3, and P65 in the WT, STING^KO^, and STING^RE^ groups were determined using ImageJ software. Data (mean ± SD) are from three independent triplicate experiments. ns, not significant; *P < 0.05; **P < 0.01; ***P < 0.001; ****P < 0.0001.

STING signaling plays a critical role in antiviral immunity by promoting interferon responses and is also a common target for viral immune evasion. To investigate the impact of this pathway on SADS-CoV infection, we activated STING using the agonist 2′3′-cGAMP. This treatment robustly induced the phosphorylation of STING at Ser366, a well-established marker of human STING activation (18). Functional experiments demonstrated that activation of the STING pathway significantly suppressed SADS-CoV replication (Fig. 4A), indicating its potent antiviral activity against SADS-CoV. To confirm this effect, we generated STING-deficient cell lines, such as HeLa-sh-STING cells (Fig. 4B) and HeLa-STING^KO^ cells (Fig. 4H) and infected them with SADS-CoV (MOI = 1). Silencing or knocking out STING markedly increased the expression of N protein (Fig. 4C and H). Transcriptional analysis demonstrated that SADS-CoV promoted the production of inflammatory factors, including ISG15 (Fig. 4D), IL-6 (Fig. 4E), CCL5 (Fig. 4F), and IL-8 (Fig. 4G), in a STING-dependent manner. Infection of HeLa-STING^KO^ and HeLa-STING^RE^ cells with SADS-CoV demonstrated that the virus-induced phosphorylation of NF-κB and IRF3 was mediated by the STING pathway (Fig. 4H and I). Collectively, these findings highlighted the critical role of STING in SADS-CoV infection and demonstrated its dual function in suppressing viral replication to exert antiviral effects and in modulating the NF-κB and IRF3 signaling pathways to increase the production of inflammatory cytokines and contribute to the antiviral immune response. The absence of STING increased viral replication and weakened immune responses, highlighting the role of this pathway in antiviral defense mechanisms.

### C176 promotes SADS-CoV replication in neonatal mice

**Fig 5:**
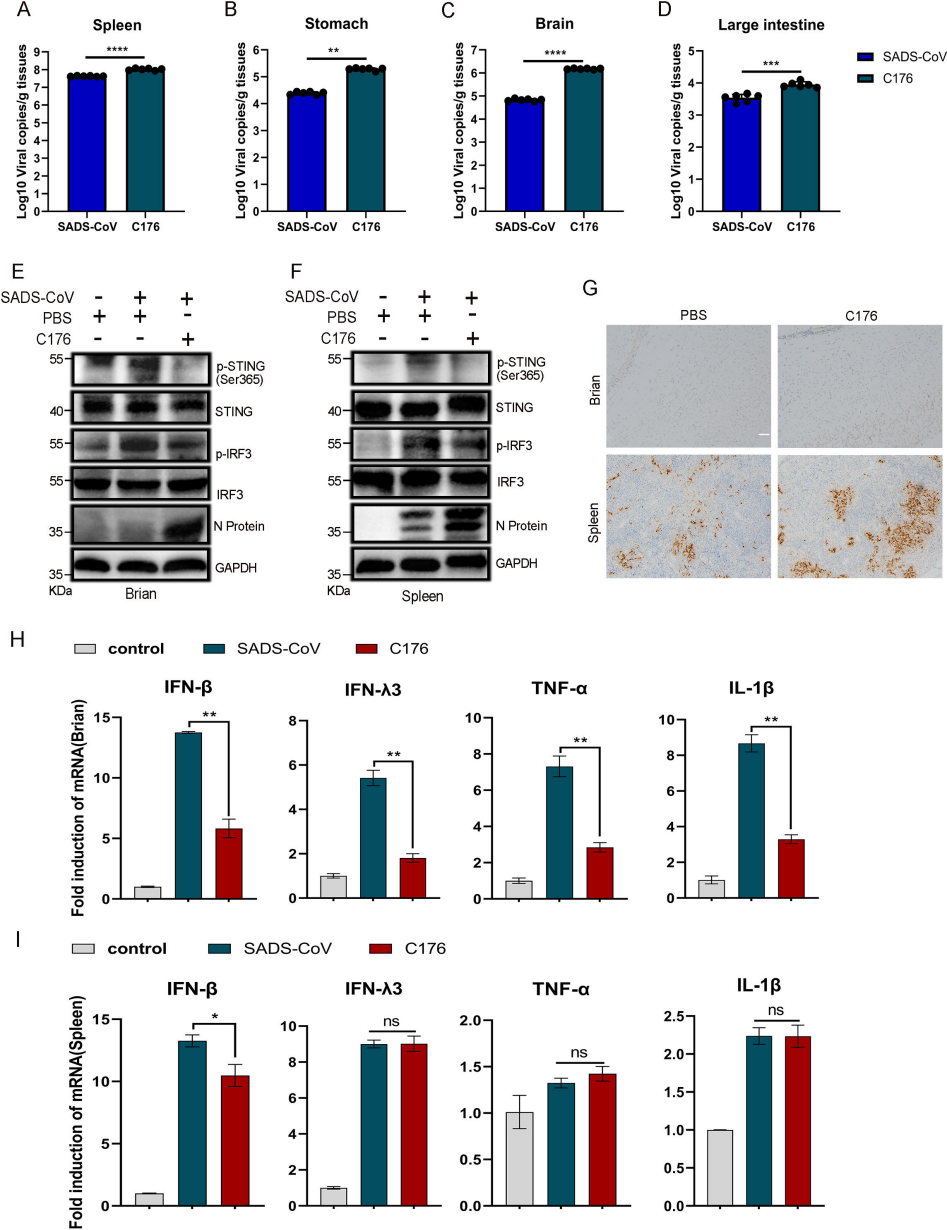
C176 promotes SADS-CoV replication in mice. SADS-CoV infection and mock infection were performed on 3-day-old neonatal mice (*n* = 6). The mice were intraperitoneally injected (12 h post-viral infection) with corn oil containing 750 nmol C176 (an inhibitor of the STING pathway) or DMSO dissolved in corn oil once per day for 3 days. The mice were euthanized after 3 days, and the brain, stomach, intestine, and spleen tissues were harvested for analysis. Real-time PCR was employed to quantify the SADS-CoV genome copy numbers in these tissues (**A–D**). Data (mean ± SD) are from three independent triplicate experiments. ns, not significant; *P < 0.05; **P < 0.01; ***P < 0.001; ****P < 0.0001. Western blotting was used to assess the phosphorylation levels of STING and IRF3 in brain (**E**) and spleen (**F**) tissues. (**G**) Immunohistochemistry was used to investigate the expression of SADS-CoV N protein in brain and spleen tissues. Scale bar: 100 μm. Real-time PCR was used to measure the expression levels of the cytokines IFN-β, IFN-λ3, TNF-α, and IL-1β in brain (**H**) and spleen (**I**) tissues. Data (mean ± SD) are from three independent triplicate experiments. ns, not significant; *P < 0.05; **P < 0.01; ***P < 0.001; ****P < 0.0001.

To investigate the role of STING signaling in SADS-CoV infection in mice, we intraperitoneally injected mice with corn oil containing 750 nmol C176 (an inhibitor of the STING pathway) or DMSO dissolved in corn oil once per day for 3 days at 12 h post-viral infection. After 3 days, the mice were euthanized, and quantitative RT-qPCR analysis of the mice treated with C176 and those infected with the virus alone was conducted. The results revealed a significant increase in viral RNA in the spleen, stomach, brain, and colon tissues of the mice treated with C176 (Fig. 5A through D). Additionally, we utilized western blotting to assess the inhibitory effect of the STING pathway on the brain and spleen tissues of mice treated with C176. C176 notably decreased the phosphorylation of STING and IRF3 but significantly increased the expression of viral proteins (Fig. 5E and F). To determine the influence of C176 on viral replication, we carried out immunohistochemistry to confirm the expression levels of SADS-CoV N proteins and detected a substantial increase in viral protein expression in the brain and spleen tissues of mice treated with C176 (Fig. 5G). To investigate the immunoregulatory response of C176 to SADS-CoV infection, we utilized qPCR to evaluate the expression of inflammatory factors and interferons in mouse brain and spleen tissues. C176 markedly suppressed the expression of IFN-β in brain and spleen tissues, which indicated that C176 exacerbated SADS-CoV infection by suppressing interferon expression in the STING pathway (Fig. 5H and I). Specifically, in the brain tissue, C176 decreased the expression of IFN-λ3, TNF-α, and IL-1β (Fig. 5H), whereas the difference was not significant in the spleen tissue (Fig. 5I). These findings demonstrated the critical role of the STING pathway in SADS-CoV infection in mice.

### DiABZI inhibits SADS-CoV replication

**Fig 6:**
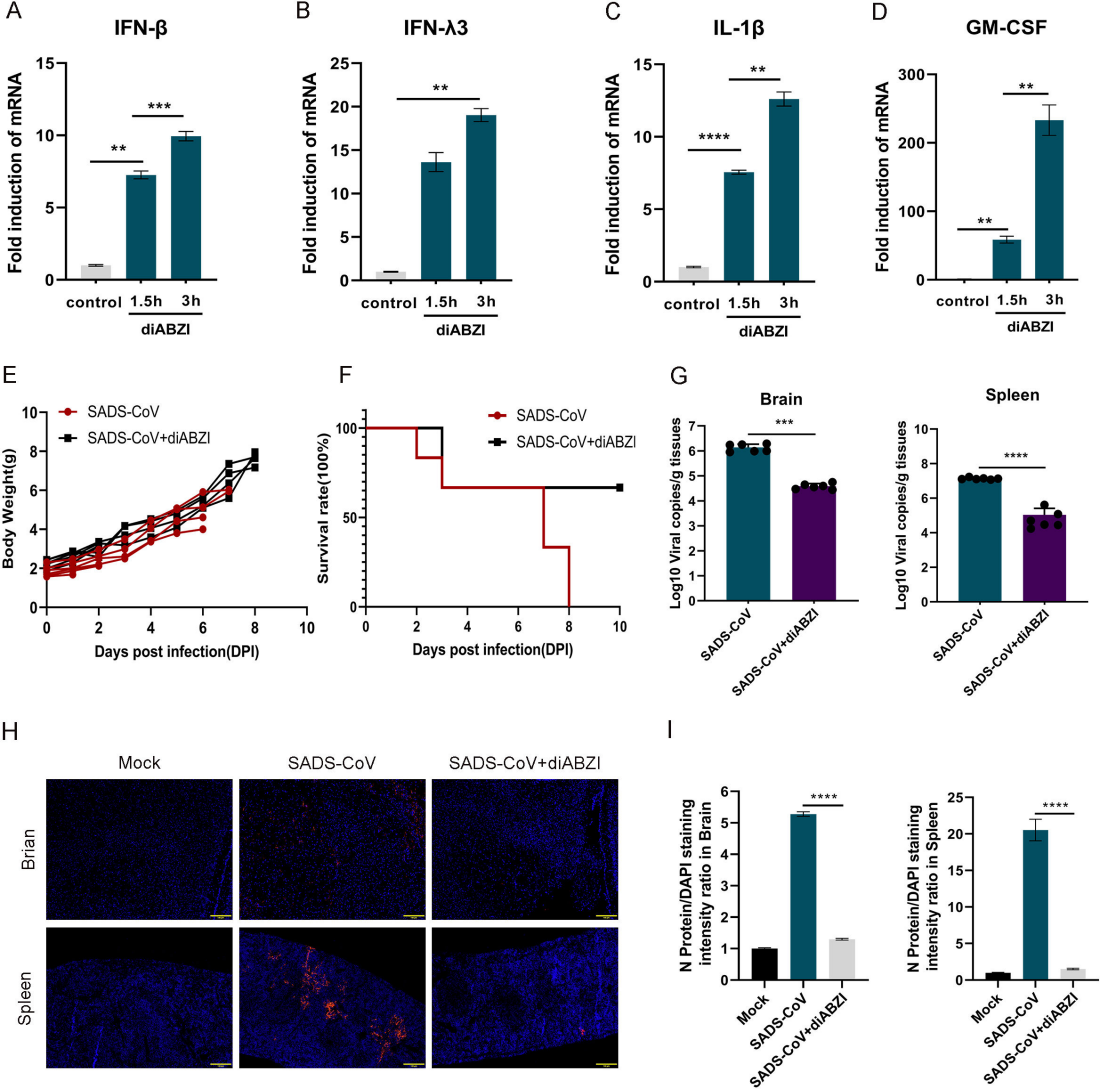
The diABZI inhibits SADS-CoV replication in mice. Neonatal mice were intraperitoneally injected with corn oil containing 2.5 mg/kg diABZI (an agonist of the STING pathway) or DMSO dissolved in corn oil, and samples were collected at 1.5 and 3 h after euthanasia. Spleen tissues were collected for real-time PCR to analyze the expression levels of the cytokines IFN-β (**A**), IFN-λ3 (**B**), IL-1β (**C**), and GM-CSF (**D**). Data (mean ± SD) are from three independent triplicate experiments. ns, not significant; *P < 0.05; **P < 0.01; ***P < 0.001; ****P < 0.0001. SADS-CoV was used to infect and mock-infect 3-day-old neonatal mice, followed by intraperitoneal injection (12 h post-viral infection) of corn oil supplemented with 2.5 mg/kg diABZI (an agonist of the STING pathway) or DMSO dissolved in corn oil once per day. Mouse body weight (**E**) and mortality rate (**F**) were recorded daily (*n* = 6). (**G**) Real-time PCR was employed to quantify the SADS-CoV genome copy numbers in these tissues.Data (mean ± SD) are from three independent triplicate experiments. ns, not significant; *P < 0.05; **P < 0.01; ***P < 0.001; ****P < 0.0001. Scale bar: 100 μm. (**H**) N protein expression in spleen tissues was assessed via immunofluorescence staining. (**I**) Quantitative analysis of N protein fluorescence (red) and cytoplasmic DAPI staining (for each sample containing at least 200 cells) was conducted. Data (mean ± SD) are from three independent triplicate experiments. ns, not significant; *P < 0.05; **P < 0.01; ***P < 0.001; ****P < 0.0001.

To investigate the efficacy of antiviral drugs in limiting the replication of SADS-CoV, we assessed the ability of diABZI, a STING pathway agonist, to provide protection against RNA viruses. Intraperitoneal injection of diABZI (2.5 mg/kg) *in vivo* resulted in the upregulation of IFN-β, IFN-λ3, IL-1β, and GM-CSF transcription (Fig. 6A through D). The mice experienced weight loss and mortality starting on the second day after infection with SADS-CoV (Fig. 6E and F). However, C57BL/6J mice that received a single intraperitoneal injection of diABZI did not lose weight (Fig. 6E) or die on the second day (Fig. 6F) after SADS-CoV infection. To further characterize the antiviral activity of diABZI, viral genome copy numbers in the brains and spleens of treated mice were quantified by qPCR. A significant reduction in viral load was observed in both tissues (Fig. 6G). Immunofluorescence staining was subsequently performed to assess the expression of the viral N protein in the spleen tissue. Compared with the control treatment, diABZI markedly reduced N protein expression (Fig. 6H and I). Taken together, these results demonstrated that diABZI effectively suppressed SADS-CoV replication *in vivo*.

### Conserved antiviral activity of diABZI across species

**Fig 7:**
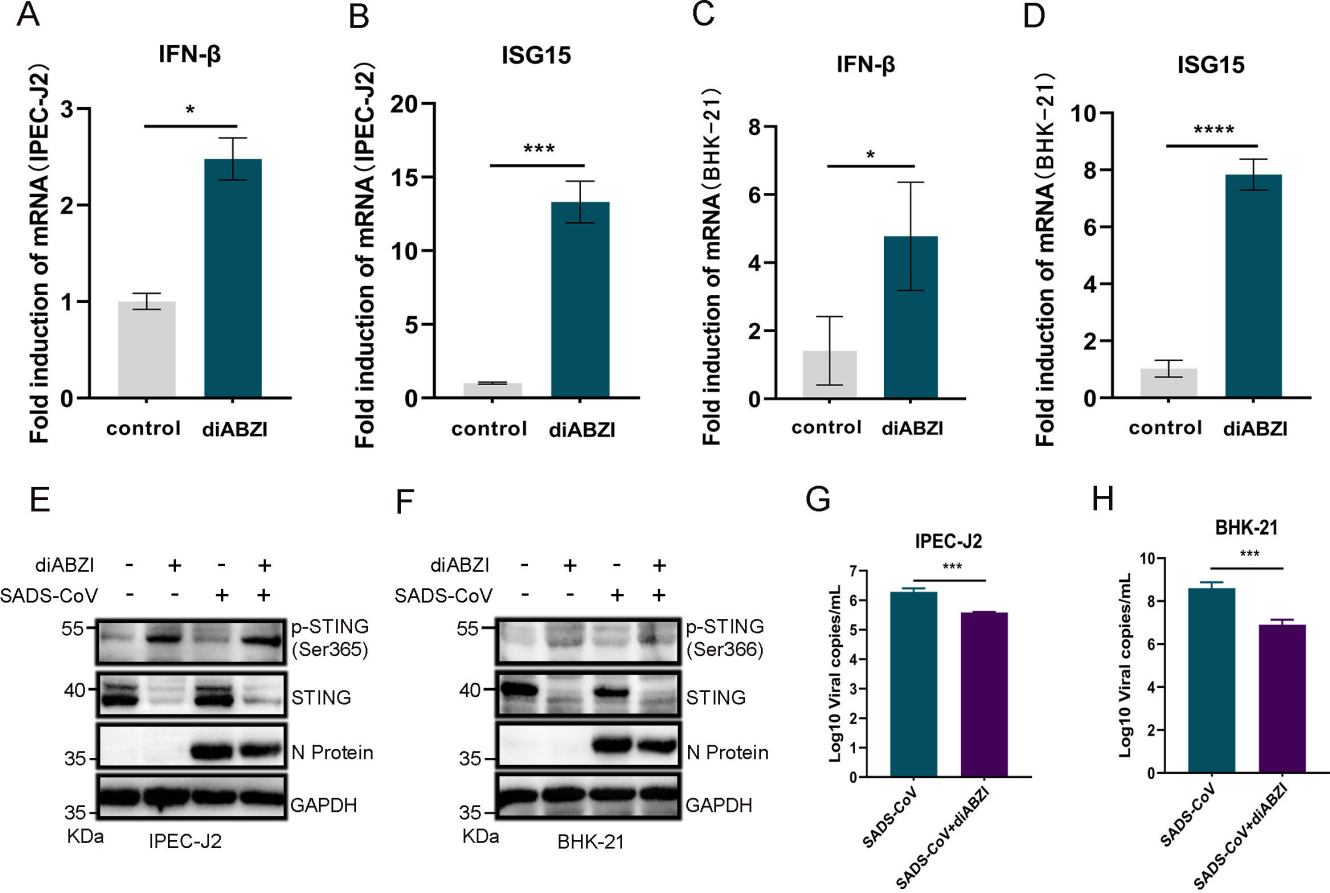
diABZI activates the STING pathway and inhibits SADS-CoV replication in porcine and rodent cells. (**A–D**) IPEC-J2 and BHK-21 cells were treated with diABZI (1 μM) or DMSO for 12 h. mRNA expression levels of IFN-β (**A, C**) and ISG15 (**B, D**) were measured by RT-qPCR and normalized to GAPDH. Data (mean ± SD) are from three independent triplicate experiments. ns, not significant; *P < 0.05; **P < 0.01; ***P < 0.001; ****P < 0.0001. (**E, F**) IPEC-J2 (**E**) and BHK-21 (**F**) cells were pretreated with diABZI (1 μM) for 6 h, followed by infection with SADS-CoV at an MOI of 1 for 24 h. Phosphorylation of STING (p-STING) and viral nucleocapsid (N) protein expression were analyzed by immunoblotting. (**G, H**) Viral genomic RNA levels in infected IPEC-J2 (**G**) and BHK-21 (**H**) cells were quantified by RT-qPCR and expressed as SADS-CoV genome copies. Data (mean ± SD) are from three independent triplicate experiments. ns, not significant; *P < 0.05; **P < 0.01; ***P < 0.001; ****P < 0.0001.

Having demonstrated the *in vivo* efficacy of diABZI in a murine model of lethal SADS-CoV infection, we next sought to determine whether this antiviral strategy is functional in the natural host. Given that swine served as the natural host of SADS-CoV, assessment of diABZI activity in porcine cells was essential to evaluate its potential for antiviral intervention. We therefore examined the immunomodulatory and antiviral effects of diABZI in porcine intestinal epithelial IPEC-J2 cells and SADS-CoV-permissive hamster kidney BHK-21 cells. DiABZI treatment induced strong upregulation of IFN-β and ISG15 at the transcriptional level (Fig. 7A through D). This response was accompanied by robust phosphorylation of STING, indicating activation of the STING signaling pathway in both cell types (Fig. 7E and F). Functionally, diABZI significantly suppressed SADS-CoV replication, as demonstrated by reduced viral N protein expression (Fig. 7E and F) and decreased infectious viral copy numbers (Fig. 7G and H). These results established that diABZI-mediated innate immune activation and antiviral activity were conserved between porcine and rodent cells. The cross-species efficacy supported the physiological relevance of the murine infection model and provided a rationale for the development of STING agonists as a targeted antiviral strategy in the natural host of SADS-CoV.

### The antiviral effect of diABZI was mediated through the STING pathway

**Fig 8:**
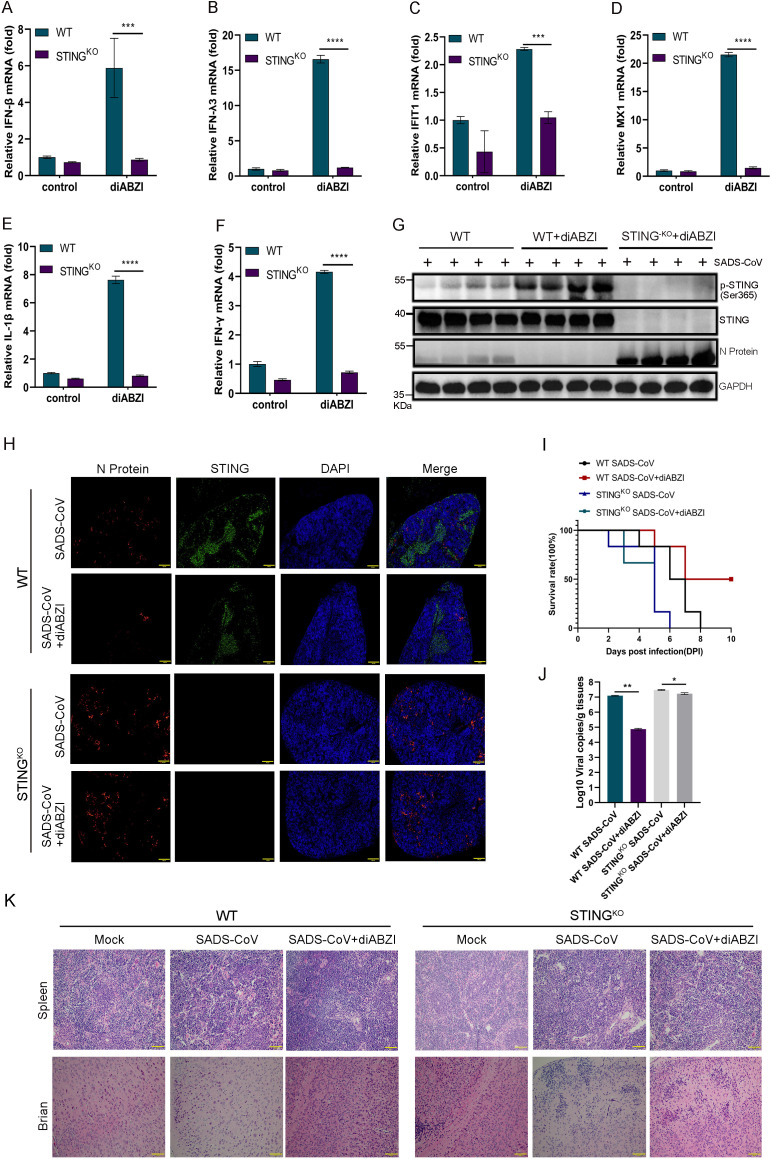
The antiviral activity of diABZI against SADS-CoV is STING dependent. SADS-CoV or mock infection of neonatal C57BL/6J wild-type (WT) and C57BL/6J Smoc-STING^KO^ (STING^KO^) 3-day-old neonatal mice (*n* = 6) was performed, followed by intraperitoneal injection (12 h post-viral infection) of corn oil containing 2.5 mg/kg diABZI (an agonist of the STING pathway) or DMSO dissolved in corn oil once per day for 3 days. The mice were euthanized after 3 days, and spleen tissues were collected for real-time PCR analysis, with a focus on the expression levels of the cytokines IFN-β (**A**), IFN-λ3 (**B**), IFIT1 (**C**), MX1 (**D**), IL-1β (**E**), and IFN-γ (**F**). Scale bar: 100 μm. Data (mean ± SD) are from three independent triplicate experiments. ns, not significant; *P < 0.05; **P < 0.01; ***P < 0.001; ****P < 0.0001. (**G**) Western blotting was used to evaluate STING and SADS-CoV N protein levels and the phosphorylation status of the STING protein in the spleen. (**H**) Immunofluorescence was used to detect the expression of STING and SADS-CoV N protein in the spleen. (**I**) Mouse mortality rates were recorded daily. (**J**) The expression of SADS-CoV genome copies in spleen tissues was assessed using RT-qPCR. Scale bar: 100 μm. Data (mean ± SD) are from three independent triplicate experiments. ns, not significant; *P < 0.05; **P < 0.01; ***P < 0.001; ****P < 0.0001. (**K**) Pathological examinations of the brain and spleen were performed via H&E staining. Scale bar: 100 μm.

The immunomodulatory activity of diABZI was evaluated in WT (C57BL/6J STING-WT, denoted WT) and STING-deficient (C57BL/6J Smoc-STING^KO^, denoted STING^KO^) mice. Following intraperitoneal administration of diABZI (2.5 mg/kg), significant upregulation of IFN-β, IFN-λ3, IFIT1, Mx1, IL-1β, and IFN-γ expression was observed in WT mice (Fig. 8A through F). This induction was not detected in STING^KO^ mice, indicating that diABZI-dependent immune activation required a functional STING pathway. Phosphorylation of STING was detected in WT mice after treatment but was absent in STING^KO^ mice (Fig. 8A through F). Suppression of SADS-CoV N protein expression was observed in the spleen tissues of diABZI-treated WT mice (Fig. 8G and H). In contrast, no reduction in N protein levels was detected in STING^KO^ mice, despite treatment. Viral antigen expression was also higher in the untreated STING^KO^ mice than in the WT control mice. In SADS-CoV-infected mice, diABZI treatment significantly improved survival and reduced the number of viral genome copies in the spleens of WT mice (Fig. 8I and J). However, this protective effect was completely lost in STING^KO^ mice, in which all infected mice succumbed to infection and viral replication remained high. Histopathological analysis revealed that the spleen damage induced by infection was alleviated in WT mice after diABZI administration, but no improvement was observed in STING^KO^ mice regardless of treatment (Fig. 8K).

### The protective effect of diABZI involved both type I interferon-dependent and -independent mechanisms

**Fig 9:**
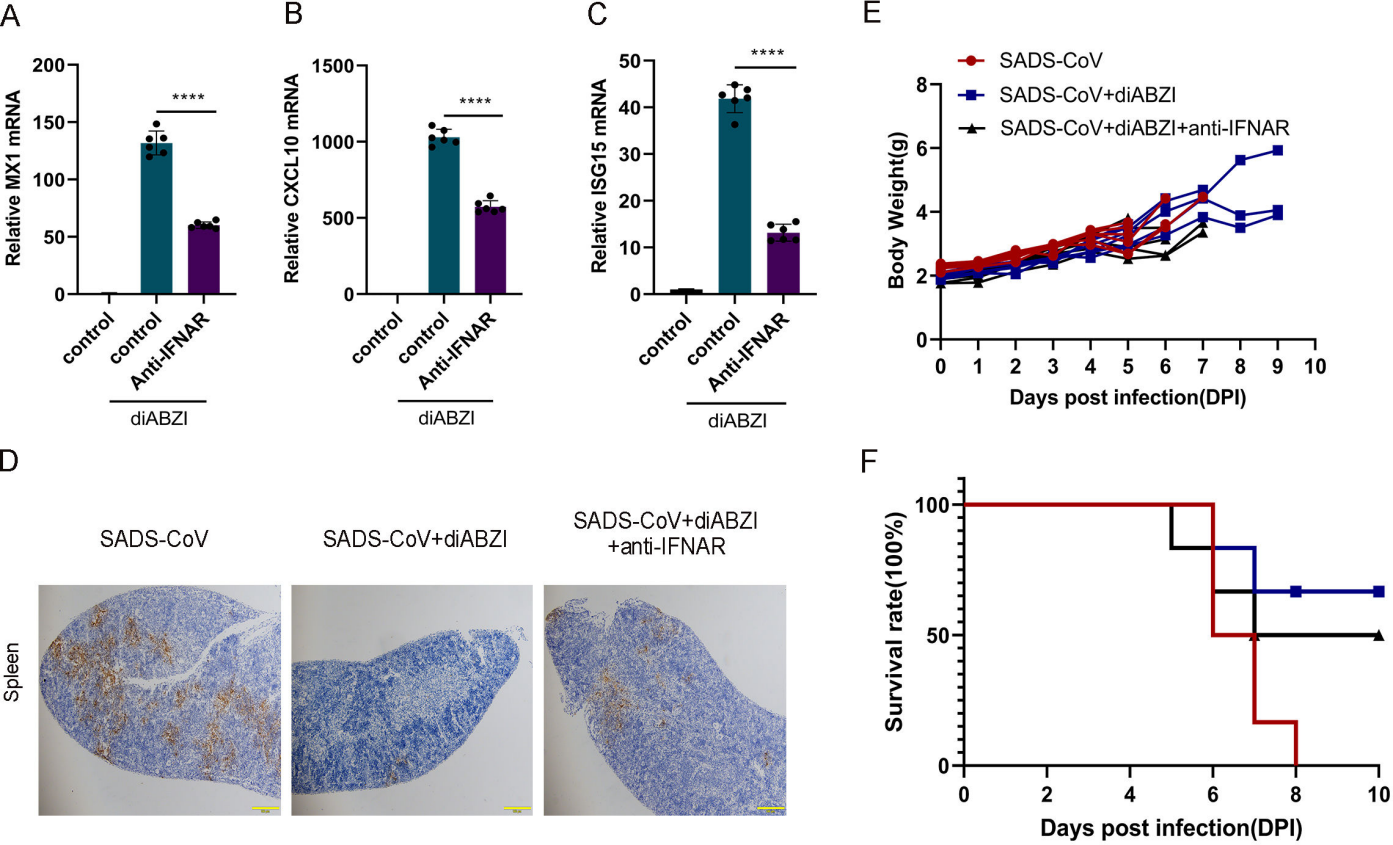
The diABZI protects mice from SADS-CoV-induced damage by promoting IFN production. Neonatal C57BL/6J wild-type (WT) mice were intraperitoneally administered IgG1 + diABZI (*n* = 6) or anti-IFNAR (1 mg) + diABZI (*n* = 6). Spleen tissues were collected for qPCR analysis to assess the mRNA expression levels of the interferon-stimulated genes MX1 (**A**), CXCL10 (**B**), and ISG15 (**C**). Data (mean ± SD) are from three independent triplicate experiments. ns, not significant; *P < 0.05; **P < 0.01; ***P < 0.001; ****P < 0.0001. Additional neonatal WT mice received the same treatments followed by infection or mock infection with SADS-CoV. Immunohistochemistry was performed to detect SADS-CoV nucleocapsid (N) protein expression in tissues (**D**). Mouse body weight loss (**E**) and survival (**F**) were monitored daily. Scale bar: 100 μm.

To assess the contribution of type I interferon signaling, IFNAR was blocked by intraperitoneal injection of neutralizing antibodies. The diABZI-induced expression of ISGs, including Mx1, CXCL10, and ISG15, was significantly suppressed (Fig. 9A through C), confirming effective pathway inhibition. In infected WT mice, IFNAR blockade partially reversed the antiviral effect of diABZI. N protein expression in spleen tissue was partially restored (Fig. 9D), and the survival benefit was reduced. However, complete abrogation was not observed: mortality was delayed, and body weight loss was attenuated compared with those in the control groups (Fig. 9E and F). These results indicated that while type I interferon signaling contributed to diABZI-mediated protection, interferon-independent mechanisms also played a role.

## DISCUSSION

SADS-CoV is an emerging bat coronavirus that induces severe acute diarrhea and dehydration in piglets. As a zoonotic virus, SADS-CoV has potential for cross-species transmission and thus has concerning public health implications (19). First, SADS-CoV was identified in Guangdong Province, China (20), but the virus has since been reported in Fujian (21), Jiangxi, and Guangxi (22). In May 2021, a fatal outbreak of SADS-CoV occurred at a large-scale pig farm in Guangxi and resulted in the death of more than 3,000 piglets (23). Currently, there are no approved vaccines or antiviral therapies available for SADS-CoV infection, but effective countermeasures are urgently needed (24). *In vivo* studies of SADS-CoV have relied on piglet models, which have high costs and technical complexities. In contrast, murine models offer several advantages, including lower costs, ease of handling, and simplified experimental procedures, and previous research has shown that SADS-CoV can infect neonatal mice through intragastric and intraperitoneal inoculation and lead to subclinical manifestations (8, 9). However, studies utilizing murine models for SADS-CoV research are limited. In this study, we sought to describe the pathogenesis of SADS-CoV in mice and evaluate potential therapeutic strategies. After intraperitoneal SADS-CoV injection, WT C57BL/6J mice presented severe clinical symptoms, such as weight loss, spasms, and a high mortality rate. The mortality of 3-day-old mice was 100%, whereas the mortality of 7-day-old mice decreased to 16.7%. These findings aligned with the high pathogenicity of SADS-CoV infection that was previously demonstrated for piglets under 5 days of age (25). This discovery, which is consistent with research on coronavirus infections, highlights the variation in the immune response across mice of different ages (26), possibly due to the weaker immune defenses in neonatal mice.

SADS-CoV infection induced pathological damage to multiple organs in mice, which was consistent with the findings of prior investigations (8, 9). In the present study, SADS-CoV-infected mice displayed watery diarrhea and concurrent neuroinflammation. The detection of viral RNA in brain tissues following intraperitoneal inoculation indicated hematogenous or neural dissemination, raising the possibility of broader tissue tropism than previously recognized. The high variability in viral RNA levels observed in the heart and stomach at 7 dpi (Fig. 1D) primarily reflected significant interindividual differences in host response. Such variability underscores the complex and individual-specific nature of SADS-CoV pathogenesis in these tissues. Similarly, porcine epidemic diarrhea virus and feline infectious peritonitis virus, which are transmitted primarily via the gastrointestinal tract, also trigger neuroinflammation in the brain tissues of infected mice (27, 28). Despite administering the virus via intraperitoneal injection, viral replication was still evident in the brain tissues of mice, indicating the potential for variation in tissue tropism and clinical presentation of SADS-CoV across different hosts. Although intraperitoneal inoculation does not mimic the natural route of SADS-CoV infection, it offers a reproducible and well-controlled method for establishing systemic infection in neonatal mice, enabling a focused analysis of viral pathogenesis and host immune responses during disseminated disease.

While STING has been traditionally associated with cytosolic DNA sensing during DNA virus infection, accumulating evidence has indicated its involvement in host defense against RNA viruses, including coronaviruses. For example, SARS-CoV-2 has been shown to induce mtDNA release, leading to cGAS-dependent STING activation and subsequent type I interferon production (29). However, the role of STING in alphacoronaviruses—particularly those causing severe neonatal disease—remains largely unexplored. Our findings revealed that SADS-CoV infection activated the cGAS-STING signaling axis in neonatal mice, despite being an RNA virus. Activation of this pathway was evidenced by the phosphorylation of STING at Ser365 in multiple tissues, including the brain, spleen, and intestine, along with a distinct subcellular redistribution of STING and cytoplasmic colocalization of γH2AX and cGAS. These results suggested that SADS-CoV might trigger innate immune sensing through noncanonical mechanisms involving genomic instability or nuclear membrane disruption, leading to cytosolic DNA leakage and subsequent cGAS-STING pathway engagement. Using a neonatal mouse model that faithfully recapitulated key clinical features of SADS-CoV infection, including watery diarrhea and progressive weight loss, we further showed that the STING pathway remained functionally competent despite the inherent immaturity of the neonatal immune system. The pronounced activation of STING-IRF3 signaling and the consequent induction of interferon responses in infected pups (Fig. 3) confirmed that this innate immune axis functioned early in life and contributed to antiviral host defense.

In SADS-CoV replication, our *in vitro* experiments revealed that STING pathway activation inhibited viral replication and modulated STING-dependent inflammatory responses. STING knockout resulted in increased viral replication and weakened immune responses, which underscored the pivotal role of the STING pathway during SADS-CoV infection in cellular models. To further validate the function of the STING pathway in SADS-CoV infection *in vivo*, we utilized C176, a specific STING inhibitor. Our results revealed that STING inhibition significantly increased SADS-CoV replication in mice, which was consistent with the results of previous studies. The lower N protein signal in the brain than in the spleen might reflect differences in viral tropism and cellular permissiveness across tissues. Notably, C176 has been shown to promote viral replication in SARS-CoV-2 infection by suppressing STING-dependent interferon responses (30, 31). These observations highlight the critical role of STING in controlling coronavirus replication and suggest that its inhibition may exacerbate viral pathogenesis. Conversely, STING agonists such as diABZI are promising antiviral agents. Our study demonstrated that treatment with the STING agonist diABZI significantly suppressed SADS-CoV replication and ameliorated disease in neonatal mice. This protective effect was strictly dependent on a functional STING pathway and involved both type I interferon-dependent and interferon-independent mechanisms of antiviral defense. To our knowledge, this is the first study to demonstrate the *in vivo* therapeutic efficacy of a pharmacologically activated STING pathway against SADS-CoV infection. Moreover, it represents one of the earliest examples of host-directed antiviral intervention in a neonatal model of lethal coronavirus infection. These findings underscore the potential of STING-targeted strategies as broad-spectrum approaches against emerging coronaviruses, particularly in vulnerable neonatal populations. While diABZI has been tested in adult models of viral infection (32, 33), its successful application in neonates—a population with distinct immune dynamics and heightened susceptibility—adds a critical developmental dimension to STING-based therapeutics. Although type I interferon signaling contributed to the anti-SADS-CoV activity of diABZI, the partial protection observed in IFNAR-blocked mice suggested that additional STING-dependent mechanisms were involved. Notably, robust STING activation has been shown to induce gasdermin D-mediated pyroptosis (34) and promote autophagy (35), two noncanonical innate immune pathways with established antiviral functions. The potential contribution of these pathways to diABZI-mediated control of SADS-CoV infection warrants further investigation.

Although diABZI demonstrates significant antiviral efficacy, its potential immunotoxicity warrants careful consideration. Excessive activation of the STING pathway is increasingly linked to inflammatory pathologies (36, 37). Notably, diABZI has been shown to trigger PANoptosis—a highly proinflammatory form of programmed cell death—and contribute to the development of ARDS in murine models (38). These observations highlight the critical importance of dosage and timing in minimizing immunopathological risk. In our study, a low dose of diABZI was administered early post-infection, effectively suppressing viral replication without evidence of hyperinflammation or tissue injury during the observation period. These findings suggested that precise modulation of STING activity through timing, dosage, and delivery strategies could achieve a favorable balance between antiviral protection and immunopathological risk. Approaches such as pulsed low-dose regimens, nanoparticle-mediated targeted delivery, and definition of the therapeutic window will be key to clinical translation (39, 40). Furthermore, given the species-specific differences in STING biology, the direct translation of therapeutic efficacy from mouse models to pigs, the natural host of SADS-CoV, remained a hypothesis requiring validation (38). However, this study demonstrated that diABZI effectively activated the STING pathway and suppressed viral replication in porcine IPEC-J2 cells, which directly countered the species-specific barrier and provided critical functional evidence for such translational research. Thus, our work not only confirmed the antiviral potential of STING-targeted strategies but also built a crucial bridge for advancing proof-of-concept studies in pig models.

## MATERIALS AND METHODS

### Cell culture and virus

HeLa (ATCC, CCL-2), HeLa-STING^KO^ (STING^KO^), HeLa-STING^RE^, Vero (ATCC, CRL-1586), IPEC-J2 (ZY1304, BioGenesis), and BHK-21 (ATCC, CCL-10) cells were maintained in DMEM (Gibco, USA) supplemented with 10% FBS (Gibco, USA). The SADS-CoV virus was propagated, and viral titers were determined in Vero cell lines in maintenance DMEM supplemented with 10 μg/mL trypsin (Gibco, USA).

### Mouse infection experiments

WT C57BL/6J mice of different ages and C57BL/6J Smoc-STING^KO^ mice were procured from Shanghai Model Organisms Center, Inc. The mice were housed at the Wenzhou University Laboratory Animal Center. The mice were intraperitoneally inoculated with a 10^6^ TCID_50_ dose of the virus, whereas the control mice received an equivalent volume of medium. The weight, clinical manifestation of symptoms, and survival rate of the mice were monitored daily until 14 dpi. The scoring criteria for clinical symptoms of SADS-CoV infection in mice are shown in Table S1.

### Viral load determination

To assess the viral load, mouse tissue samples were homogenized in PBS, and viral RNA was extracted from the homogenized tissue with an RNA extraction kit (Sangon Biotech, China). qPCR analysis targeting the SADS-CoV N gene was performed to detect viral RNA. The primers used are listed in Table S2.

### Real-time PCR analysis

To quantify cytokines and IFNs, RT-qPCR was conducted using GoTaq qPCR Master enzyme premix (Promega, USA) for SYBR fluorescence detection. The reaction mixture was initially incubated at 95°C for 5 min, followed by 40 cycles of denaturation at 95°C for 10 s and annealing/extension at 60°C for 30 s with a QuantStudio 3 Real-Time PCR System (Thermo Fisher Scientific, USA). The primers used are shown in Table S2. The expression of the target gene was normalized to that of endogenous GAPDH, and the relative expression was determined via the 2^−ΔΔCT^ method.

### Histopathological and immunohistochemical analyses

SADS-CoV-infected mice were anesthetized at specified times. The mouse tissues were fixed in 4% paraformaldehyde, embedded in paraffin, and sectioned. For H&E staining, the tissue sections were dewaxed, dehydrated, and stained with hematoxylin for nuclei and eosin for cytoplasm, after which the results were observed. For immunohistochemistry, the tissue sections were dewaxed, dehydrated, incubated with SADS-CoV N antibody in a wet box for 1 h, incubated with an HRP-labeled rabbit secondary antibody (Bioss, China) for 30 min at room temperature, incubated with DAB (Beyotime, China) for color development, counterstained with hematoxylin (Beyotime, China), and observed under a microscope (Olympus, Japan).

### Immunofluorescence assay

Paraffin sections of mouse tissues were dewaxed and dehydrated, fixed with 95% alcohol for 30 min, and incubated with STING and SADS-CoV N antibodies in a wet box for 2 h. After being washed with PBS, the sections were incubated with Alexa Fluor 594-conjugated and Alexa Fluor 488-conjugated (Cell Signaling Technology, USA) fluorescent secondary antibodies in the dark for 1 h. After the samples were washed, anti-fluorescence quenching sealing solution containing DAPI (Beyotime, China) was added to stain the nuclei, and the samples were observed under a fluorescence microscope (Olympus, Japan).

### Western blotting

Mouse tissues or cells were collected, and the lysate supernatant was processed with loading buffer (Takara, Japan) for western blotting. The samples were separated by SDS-PAGE, the proteins were transferred to PVDF membranes for incubation with primary and secondary antibodies, and the protein bands were visualized using a chemical chromogenic method (NCMbiotech, China).

### Statistics

Statistical analyses were conducted using GraphPad Prism 9 (GraphPad Software, USA). Two-tailed Student’s *t*-tests were used to compare two groups, and significant differences were determined as follows: ns, not significant; *P < 0.05; **P < 0.01; ***P < 0.001; and ****, *P* < 0.0001.

## Data Availability

The original contributions in the study are presented in the supplementary material, including all the western blot images. Further inquiries can be directed to the corresponding authors.

## SUPPLEMENTAL MATERIAL

Supplemental tables jvi.01703-25-s0001.docx Table S1, clinical scores of SADS-COV-infected mice; Table S2, primers used for cloning and qPCR.

ASM does not own the copyrights to Supplemental Material that may be linked to, or accessed through, an article. The authors have granted ASM a non-exclusive, world-wide license to publish the Supplemental Material files. Please contact the corresponding author directly for reuse.
