# Supplementary material for: STING agonist diABZI confers protection against swine acute diarrhea syndrome coronavirus in neonatal mice by activating antiviral immunity
Source: J Virol. 2025 Dec 29;100(2):e01703-25. doi: 10.1128/jvi.01703-25 (PMC12911889; doi:10.1128/jvi.01703-25)
Supplement: Supplemental tables — Table S1, clinical scores of SADS-COV-infected mice; Table S2, primers used for cloning and qPCR. [file jvi.01703-25-s0001.docx]

**STING Agonist diABZI Confers Protection against Swine Acute Diarrhea Syndrome Coronavirus in Neonatal Mice by Activating Antiviral Immunity**

Yuying Li^a,^d, Wei Chen^b^, Xinyu Zhang^c^, Jiyong Zhou^a^, Yanqing Hu^b^, Yimin Zhou^b^, Tian Lan^b^, Haixin Huang^b^, Lulu Xie^b^, Yan Qin^b^, Lin Zhou^b^, Wenchao Sun^b*^, Huijun Lu^a,d*^

^a^MOA Key Laboratory of Animal Virology, Zhejiang University Center for Veterinary Sciences, Hangzhou 310058, China.

^b^Wenzhou Key Laboratory for Virology and Immunology, Institute of Virology, Wenzhou University, Wenzhou 325035, China.

^c^Agricultural College, Yanbian University, Yanji 133000, China.

^d^Changchun Veterinary Research Institute, Chinese Academy of Agricultural Sciences, Changchun 130122, China

*Corresponding author:

Prof. Huijun Lu

Email addresses: huijun_lu@126.com

Associate Prof. Wenchao Sun

Email addresses: sunwenchao131@163.com

The author order was determined based on seniority.

**Supplementary Tables**

**S1 Table. Clinical score of SADS-COV infected mice**

| Score | | | Description | Key Symptoms |
| --- | --- | --- | --- | --- |
| 0 | No clinical signs | | Healthy, no signs of distress or weight loss | |
| 1 | Mild signs | | Lethargy and inactivity,slight weight loss(<10%) | |
| 2 | Moderate signs | | Mild diarrhea, weight loss (10-15%) | |
| 3 | Severe signs | | Watery diarrhea, weight loss (15-20%) | |
| 4 | Very severe signs | | loss of coordination, weight loss(>20%) | |
| 5 | Critical/terminal signs | | Near-death state, no movement, moribund state | |

**S2 Table. Primers used for cloning and qPCR**

| Primer name | Forward sequence (5'–3') | Reverse sequence (5'–3') |
| --- | --- | --- |
| SADS-CoV-N | TTGAACAGGCGGAATCTCG | AAAAGTGCCAGTTAGAAGG |
| M-IFN-β | CCAGCACTGGGTGGAATGAG | AGTTGAGGACATCTCCCACG |
| M-GAPDH | CTACCCCCAATGTGTCCGTC | TGAAGTCGCAGGAGACAACC |
| M-IL-1β | ATCTCGCAGCAGCACATCAA | ACGGGAAAGACACAGGTAGC |
| M-TNF-α | AGCCGATGGGTTGTACCTTG | ATAGCAAATCGGCTGACGGT |
| M-IL6 | CACTTCACAAGTCGGAGGCT | CTGCAAGTGCATCATCGTTGT |
| M- Mx1 | GACTTGCTCTTTCTGAAAAGC | GACCATAGGGGTCTTGACCA |
| M-IFIT1 | GCCTATCGCCAAGATTTAGAT | TTCTGGATTTAACCGGACAGC |
| M-IFN-γ | CAGGCCATCAGCAACAACAT | ACCTGTGGGTTGTTGACCTC |
| M-GM-CSF | GCTCACCCATCACTGTCACC | GCGGGTCTGCACACATGTTA |
| M-IFN-λ3 | AGAACCCAAGCTGACCCTGT | GCACCCTTGGCCTTTTTGAA |
| M-ISG15 | TGGGGGGTAACGATTTCCTG | CCTCATAGATGTTGCTGTGG |
| H-STING | TAGGAGAGCCACCAGAGCA | GGGAGGGAGTAGTAGAAAT |
| H-IL-6 | ACTCACCTCTTCAGAACGAATTG | CCATCTTTGGAAGGTTCAGGTTG |
| H-IL-8 | TTTTGCCAAGGAGTGCTAAAGA | AACCCTCTGCACCCAGTTTTC |
| H-GAPDH | TCTGCTCCTCCTGTTCGACAG | CCCAATACGACCAAATCCGTT |
| H-ISG15 | GAGAGGCAGCGAACTCATCT | CTTCAGCTCTGACACCGACA |
| H-CCL5 | CCCAGCAGTCGTCTTTGTCA | TACAGCAACCATGAGTACAA |
| p-GAPDH | AGCAACAGGGTGGTGGACCT | CTGGGATGGAAACTGGAAGT |
| P- IFN-β | CATCCTCCAAATCGCTCTCC | ACATGCCAAATTGCTGCTCC |
| P-ISG15 | CAGGTGTGTCAGCAGGAGGG | GCTCATCCTCCATGGGCTTC |
